# Are Anomalous Invasion Speeds Robust to Demographic Stochasticity?

**DOI:** 10.1371/journal.pone.0067871

**Published:** 2013-07-16

**Authors:** Elizabeth C. Elliott, Stephen J. Cornell

**Affiliations:** School of Biology, University of Leeds, Leeds, United Kingdom; University of Glasgow, United Kingdom

## Abstract

Two important issues for conservation are the range expansion of species as a result of climate change and the invasion of exotic species. Being able to predict the rate at which species spread is key for successful management. In deterministic models, the invasion speed of a polymorphic population can be faster than that of any of the component phenotypes, and these “anomalous” invasion speeds persist even when the mutation rate between phenotypes is vanishingly small. Here we investigate whether the same phenomenon is observed in a model with demographic stochasticity. The model that we use is discrete in time and space and we carry out numerical simulations to determine the invasion speed of a population that has two morphs which differ in their dispersal abilities. We find that anomalous speeds are observed in the stochastic model, but only when the carrying capacity of the population is large or the mutation rate between morphs is high enough. These results suggest that only species with large population sizes, such as many insect species, may be able to invade faster if they are polymorphic than if there is only a single morph present in the population.

## Introduction

The range expansion of species either as a result of climate change or from the introduction of exotic species has important consequences for conservation management. There is increasing evidence that species are shifting their distributions as a result of climate change [1]–[4] but predicting whether these species can keep up with the rate of change or whether and by how much they lag behind is a challenge. The introduction of exotic species that then spread [5] and especially those that become pests [6] can have important consequences for biodiversity [7]. Being able to predict the rate at which they spread is key for minimising the impact these species have on ecosystems.

The rate of spread of species invasions has long been investigated using simple deterministic models [8], [9]. Since these early models more complex techniques have been developed and used to model the spread of species. These include new analytical methods such as integrodifference equations, and more computational methods such as individual-based models (IBMs) which often incorporate a greater number of parameters and include stochasticity (see [10], [11] for reviews of these developments). Deterministic models have the advantage that they are elegant and give simple predictions. However, deterministic models do not take account of the discrete nature of individuals and the unpredictable nature of demographic events, and stochastic models are expected to give more realistic results that are likely to be more useful when making decisions for conservation management.

Simple, deterministic models predict that a polymorphic species can invade at an ‘anomalous’ speed, i.e. faster than a population containing any of the constituent phenotypes on its own [12], [13]. In an earlier paper, we found anomalous speeds can occur for a simple dimorphic population with mutation at birth, diffusive motion, and Lotka-Volterra competitive interactions, when there are differences in the dispersal and establishment abilities of two morphs [13]. We found that this effect persists–i.e., the invasion speed does not tend towards the faster monomorphic speed–when the mutation rate between morphs approaches zero. This is surprising because in that limit the leading edge of the invasion front (which determines the invasion speed) contains vanishingly few individuals of the minority morph. The biological interpretation of this is unclear, so the question arises as to whether anomalous speeds occur as an artefact of the fact that densities can be arbitrarily small in the model, or if they are still present in models where species are made up of individuals. In this paper we will develop a stochastic version of the model in the hope that it will shed some light on this question.

The use of deterministic and stochastic models for predicting the rate of spread of species has highlighted some important differences. In density independent models, demographic stochasticity does not generally slow invasions [14]. This was also found by Mollison [15], who showed that linear stochastic models often give the same result as deterministic models and that speeds predicted using these provide an upper bound for the more realistic nonlinear stochastic case. Incorporating demographic stochasticity into density dependent models has provided further insight into whether stochasticity affects invasion speeds [16]–[19]. These models reach varying conclusions, with Snyder [19] finding that the addition of demographic stochasticity results in marginally slower invasions and Clark *et al*. [18] finding that adding stochasticity can turn accelerating invasions into constant speed invasions. Kot *et al*. [14] also demonstrated that, in contrast to the density independent case, the combined effect of stochasticity and density dependence can slow invasions.

Travis *et al*. [20] have also highlighted the value of using both deterministic and stochastic modelling approaches when predicting invasion speeds. They found that though both models produced similar trends, their analytical model predicted significantly higher speeds than their stochastic IBM [20]. They found that increasing the amount of stochasticity incorporated into the model, through increasing the number of age classes, widened the difference in the speed predicted by the two models. Their results revealed that looking at both stochastic and deterministic models can help to more accurately predict the speed at which a species expands its range.

A general result appearing in the literature is that density dependent models with demographic stochasticity have slower invasion speeds than their deterministic counterparts. Based on these results [14], [19], [20] we may expect that a stochastic version of our dispersal polymorphism model [13] has slower invasion speeds than the deterministic version. If this is the case then it may be that anomalous speeds do not arise in stochastic models. In this paper we investigate whether anomalous speeds are preserved in a model incorporating demographic stochasticity. We do this using a discrete time and space stochastic model. The model follows our previous work [13] so that there is a polymorphic population with morphs that differ in their dispersal abilities. We carry out numerical simulations of this model in order to determine the invasion speed of the population, to find the conditions (if any) under which anomalous speeds are produced.

## Methods

The model that we use is similar to that used in our previous work which looked at the invasion of a species that has two dispersal phenotypes [13]. That model used partial differential equations (PDEs) because they are elegant, easy to analyse, and give simple results. In this manuscript we adapt our spatially explicit general Lotka-Volterra model so that it is now discrete in time and space. We do this because the stochastic equivalence of PDEs are complex and it is more straightforward to make a link between deterministic and stochastic models that are discrete rather than continuous. We will therefore first develop a discrete time and space deterministic model and then compare the results of this model to a stochastic version.

The model has the same two phenotypes with mutation between morphs as in our previous model [13]. The phenotypes differ in their dispersal and establishment abilities, so that we have:

- an establisher morph 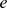 that after establishment has a higher growth rate but is a poorer disperser; and
- a disperser morph 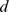 that has a lower growth rate after establishment but is a better disperser

The model simulates the invasion of these two morphs with discrete generations. Population density of the species is denoted by 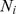 with 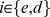 representing density of each morph. Population dynamics and dispersal then occur in discrete steps, so that in each time step the order of events occurs as follows. First, each individual of either morph produces 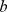 offspring, with a fraction 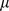 of these offspring being of the other phenotype. This gives the number of individuals of morph 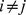 at time 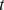 and position 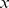 after recruitment to be
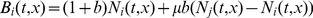
(1)


A fraction of each morph then dies. We assume a Ricker-like density dependence where the survival probability of morph 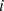 is
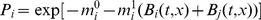
(2)


The number of individuals of morph 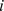 after the mortality step is then given by
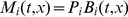
(3)


We choose density dependence to act on mortality rather than birth rate because it makes the calculations simpler, but we expect qualitatively similar results if density dependence were to act on birth. Finally a fraction 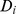 of each morph disperses, so that the number of individuals of morph 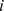 that disperse is given by
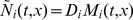
(4)


In the 1D case we have that each morph disperses with equal probability to the left and right, and in the 2D case with equal probability to the 8 neighbouring cells. This gives two discrete time and space equations that represent the population dynamics and dispersal of a polymorphic population. In 1D the iteration equation for morph 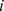 is therefore
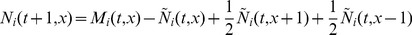
(5)


We reparameterise this model so that we can relate the parameters to our continuous time model [13], and to reduce the dimension of the parameter space we need to explore in the discrete model (see Appendix S1 in File S1 for details). We define the net growth rate 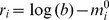 and carrying capacity 
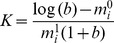
. We carry out simulations with different values of 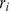 and 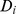 to determine the effect each morph's dispersal and establishment abilities have on the invasion speed.

Our stochastic model is an individual-based model that uses the same mean birth, death, and dispersal rates as in the deterministic model. The difference is that these are now random demographic processes, and the number of individuals 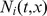 of morph 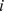 at site 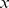 and time 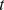 is an integer. In the recruitment step, we assume that each individual produces independently a Poisson-distributed number of offspring with mean 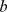, and each new birth has the same phenotype of its parent with probability 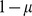 and the other phenotype with probability 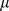. At the mortality step, we assume that each individual survives (statistically independently) with probability given by 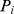 in Eqn. (2). At the dispersal step, each individual chooses (independently) to disperse with probability 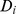, in which case it moves to a randomly chosen neighbouring site with equal probability, and otherwise remains at the same site. Since the sum of Poisson-distributed random variables also has a Poisson distribution, and the sum of Bernoulli random numbers has a binomial distribution, we can implement these individual-based processes in the model by generating Poisson-distributed pseudorandom numbers with mean given by 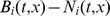 in Eqn. (1) for the total number of births of each morph at each site, and using binomial distributed pseudorandom variables to generate the total number of individuals of each morph surviving (using probability 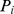 from Eqn. (2)) and dispersing (using probability 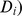 at each site. This is formally equivalent to an individual-based model, but much more computationally efficient than generating separate pseudorandom numbers for each individual.

In the stochastic model we are also interested in the effect that the carrying capacity of the population, 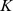, has on the invasion speed. We expect that the stochastic model should behave in a similar way to the deterministic model when 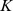 is large. It is well established that demographic fluctuations are proportional to the square root of the population size [21], so that the fluctuations are smaller relative to the population size when the population size is large. In Appendix S2 in File S1, we show explicitly how our stochastic model approaches the deterministic one when 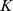 is large. When the carrying capacity is small, we expect that stochasticity will have a bigger effect on the invasion speed. We therefore ran simulations with the carrying capacity 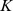 ranging from 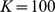 to 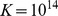 whilst keeping the other parameters constant as a way of looking at how stochasticity affects the invasion speed.

In the stochastic case we are also interested in the effect that the mutation rate, *μ*, has on the invasion speed. In our continuous model [13] we found the surprising result that, when the mutation rate is small, changing it has no effect on the invasion speed. We therefore vary the mutation rate in both models in the present manuscript to see if we observe the same result. We predict, in contrast to our previous model, that changing the mutation rate will affect the speed, in particular that the smaller the mutation rate the bigger the effect that stochasticity will have on determining the invasion speed.

We consider invasions of an introduced species into a previously unoccupied landscape in both a one and two-dimensional landscape, with half the landscape initially occupied by the population at its stable equilibrium density and half the landscape initially unoccupied. We analysed this model using both semi-analytical and simulation techniques. A prediction for the invasion speed for the deterministic model was computed by using the method of front propagation [22], which led to equations that then were solved numerically (see Appendix S3 in File S1).

Simulations of the deterministic and stochastic models were then carried out in R [23], using lattices with dimensions 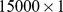 in 1D and 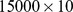 in 2D and reflecting boundary conditions at the end of the lattices. These simulations produced travelling waves which rapidly approached a constant speed as the invasion progressed. The invasion speed was estimated by calculating the distance that the density profiles at different times needed to be displaced in order to lie on top of each other. We verified that the simulations had reached a constant invasion speed by carrying these calculations out at several time points; in all cases, 10 000 time steps was sufficient for the wave speed to converge to a constant value.

In contrast to the deterministic case, the number of dimensions does matter in a stochastic model. In particular there are more fluctuations in 1D, so if we observe anomalous speeds in this case we know that the results are robust. In addition carrying out simulations in 1D is computationally cheaper than in 2D and so the results presented will be in 1D, with 2D simulations carried out to check that the same qualitative results are observed.

## Results

### Deterministic model

We first carried out deterministic simulations to investigate how the invasion speed varies with different parameter values. We compare these to the results we found in our PDE model [13]. These simulations were carried out by iterating equations (1–4) in R [24]. We carried out simulations first with each morph present in the landscape on its own and then with both morphs present. By symmetry, the deterministic model in 2D is equivalent to a 1D model, so we will only present simulation results in 1D here.

**Figure 1:**
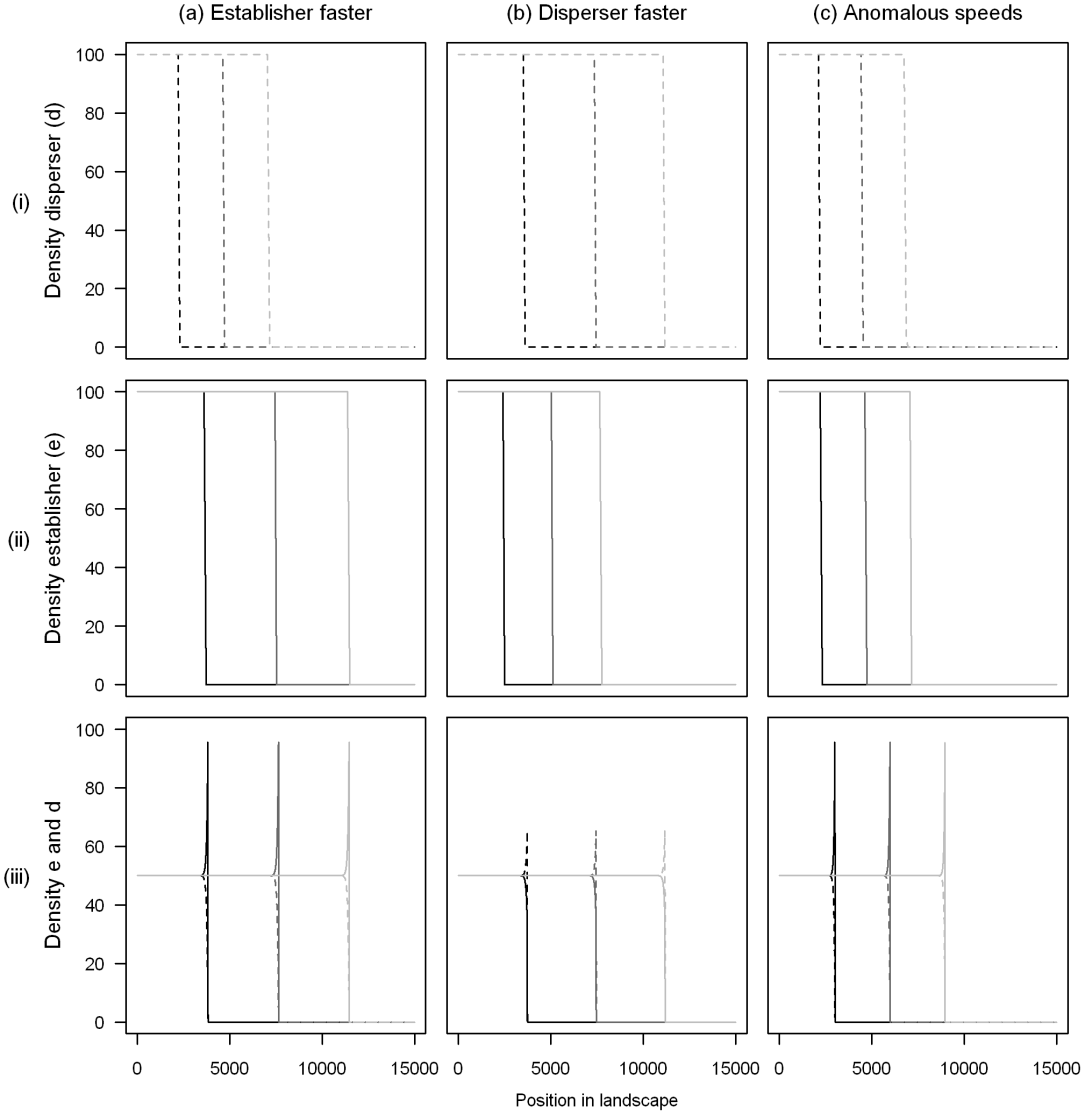
Invasion profiles of the two morphs. These show the disperser morph (dashed line) and establisher morph (solid line) when present in the landscape on their own (rows (i) and (ii)) and when mutation allows both to be present (row (iii)). The simulations were initiated with the first 100 cells occupied by each phenotype at its equilibrium population density and the remaining cells unoccupied. The simulations were run on a lattice consisting of 15000 cells. For all graphs each line represents the density profiles at a different time point, with each time point 500 units apart. In column (a) the polymorphic invasion speed is the same as the monomorphic establisher speed; in column (b) the polymorphic invasion speed is the same as the monomorphic disperser speed, and in (c) the polymorphic invasion speed is faster than either monomorphic invasion. For all simulations 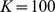, 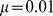, and in (a) 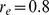, 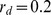, 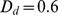, 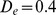; (b) 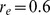, 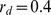, 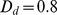, 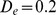; (c) 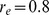, 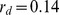, 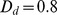, 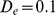.

We observe the same phenomenon as we found in our previous model [13], i.e. that there are three possible scenarios for the wavespeed (Fig. 1) depending on the relative dispersal and establishment abilities of the two phenotypes. When the dispersal abilities of the disperser and establisher are similar but the population growth rate of the establisher is much higher than that of the disperser the invasion occurs at the speed of the establisher (Fig. 1a). When the population growth rates of each morph are similar but the dispersal rate of the disperser is much higher than the dispersal rate of the establisher the invasion occurs at the speed of the disperser (Fig. 1b). However, when there is a big difference between the two phenotypes in terms of both the dispersal and establishment abilities, the invasion occurs faster than either single morph (Fig. 1c).

The main difference that we observe between the continuous and discrete time models is that with comparable parameter values all of the invasion speeds are slightly slower in the discrete version. In addition, the difference between the anomalous speed and the fastest single morph speed is not as big. For example, for the parameter values used in Fig. 1c when both morphs are present the invasion speed is 1.24 times faster than the fastest single morph, however, in the PDE model for the same ratio of dispersal and establishment rates between morphs the invasion speed is 1.38 times faster [13]. This means that a population consisting of two morphs with big differences in their establishment and dispersal abilities does not have as significant an effect on the invasion speed. Nevertheless the invasion does still occur faster than we would predict from a single morph's invasion speed.

#### Analytical calculation of invasion speed

**Figure 2:**
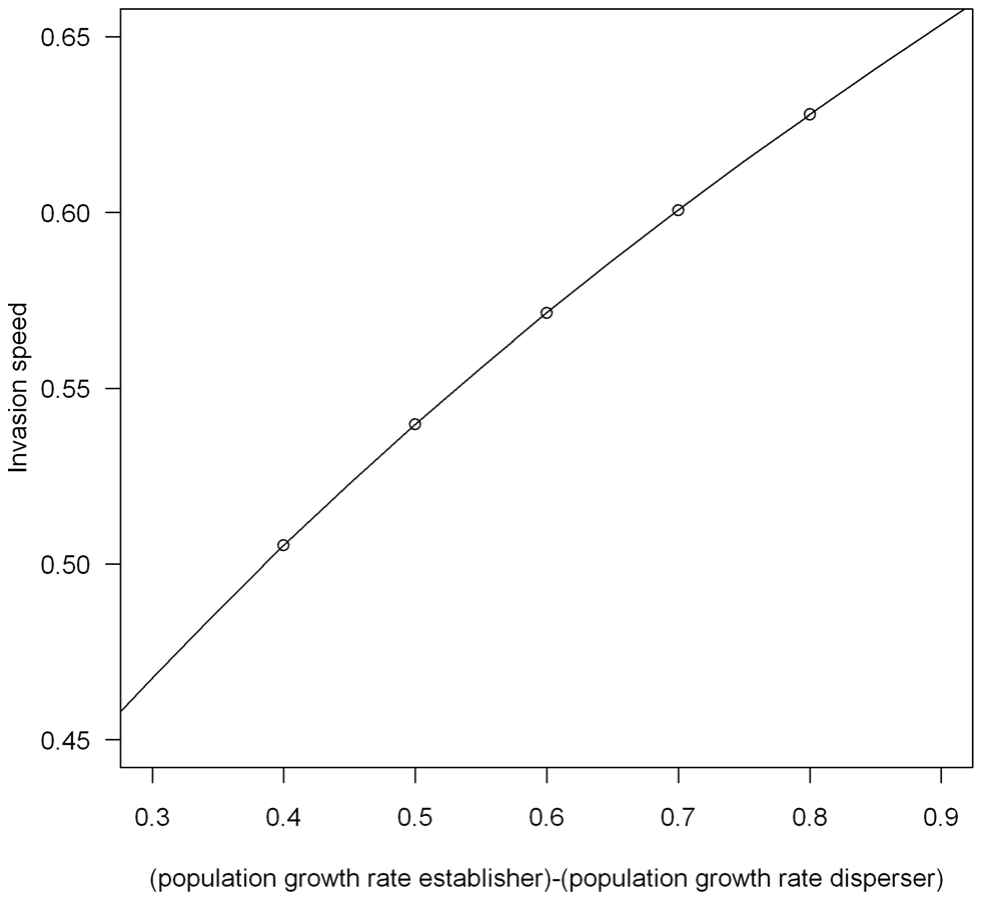
Comparison of analytical and numerical predictions of the invasion speed. This is an example of the case when polymorphism results in faster invasions than either single morph. The circles represent numerical results calculated from simulations, and the curve numerical predictions found using the van Saarloos method [27]. Parameter values used were 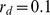, 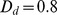, 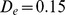, 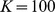, 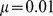 and the values of 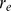 used for the numerical simulations were 0.5, 0.6, 0.7, 0.8 and 0.9.

We used the front propagation method of van Saarloos [22] to calculate the invasion speed of this polymorphic population (see Appendix S3 in File S1 for details of these calculations). This method assumes that, although the model is nonlinear, the wave speed is determined by the linear behaviour of the leading edge of the front. We find that this analytical method does indeed give the same invasion speed that we found using the numerical simulations, as can be seen in Fig. 2.

### Stochastic model

We next investigated what effect demographic stochasticity has on the invasion speed. A version of the model that includes demographic stochasticity was set up in the way described in the Model section. The model was then simulated in the same geometry as the deterministic model with simulations run on both a one and two dimensional landscape. The 2D simulations give similar predictions to the 1D and so we will only present the results of the 1D stochastic model here. We ran 10 replicates of each simulation, which proved to be a sufficient number as there was little variation between repeats. For the stochastic simulations we investigated how the carrying capacity of the population affects the invasion speed, as stochasticity is expected to have a bigger effect at low population densities. We were therefore also interested in whether there was a threshold population size for stochasticity to no longer have an effect on the invasion speed, so larger population sizes than we may realistically expect to find were investigated to show that the stochastic model converged to the deterministic model.

**Figure 3:**
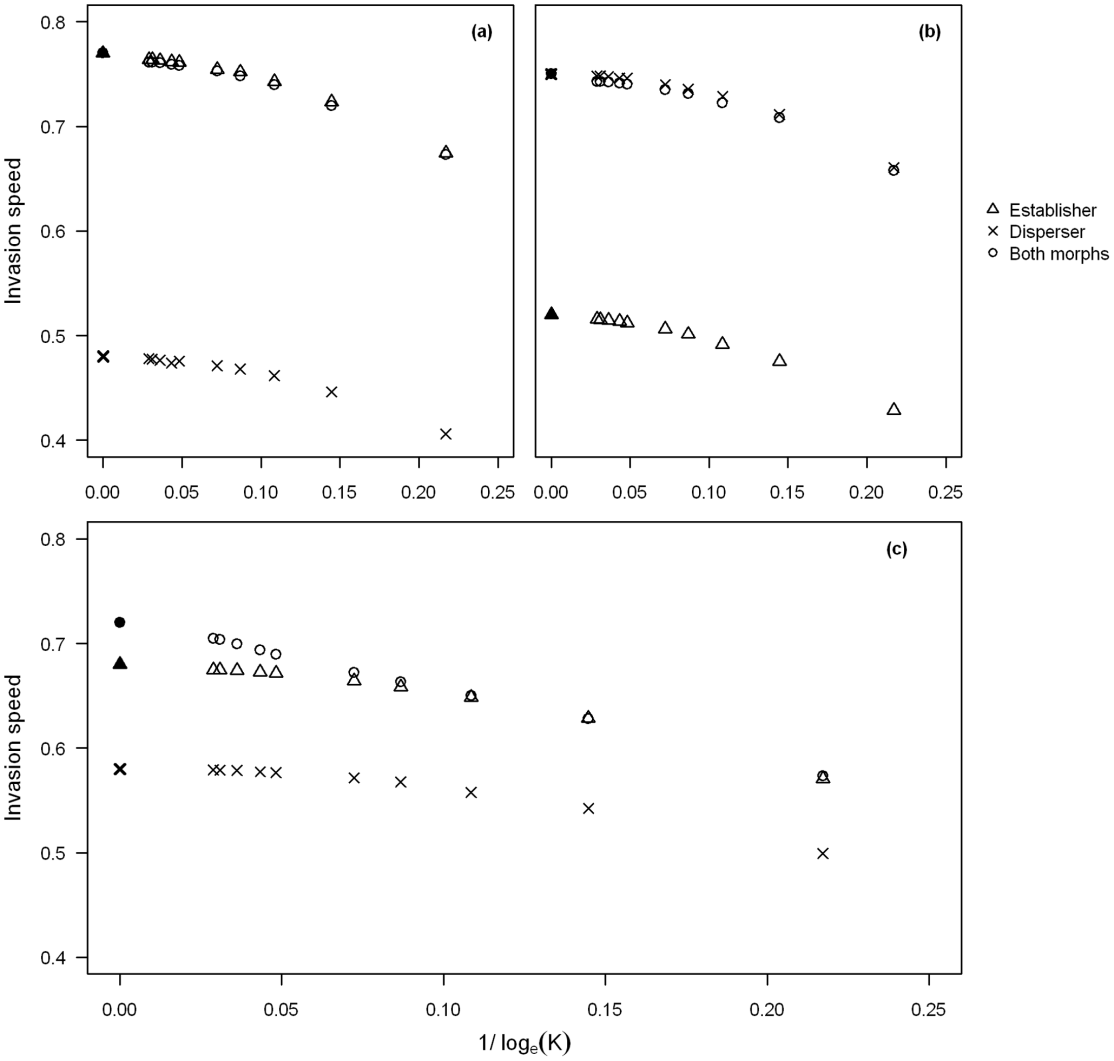
Comparison of stochastic and deterministic invasion speeds at different carrying capacities. The filled symbols represent the deterministic prediction and the empty symbols the stochastic predictions. The triangles represents the establisher morphs speed, the crosses the disperser morphs speed and the circles the invasion speed when both morphs are present. In (a) the polymorphic invasion speed is the same as the monomorphic establisher speed; in (b) the polymorphic invasion speed is the same as the monomorphic disperser speed, and in (c) the polymorphic invasion speed is faster than either monomorphic invasion. Parameter values used in (a) and (b) are the same as in Fig. 1 (a) and (b) and in (c) the parameter values used were 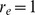, 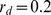, 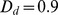, 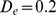, 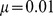, with 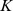 ranging from 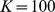 to 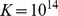.

We found for all parameter values that having a finite carrying capacity results in the invasion occurring more slowly than the deterministic prediction. Demographic stochasticity does therefore result in slower invasions as has previously been found [19], [20]. However, we find that as the carrying capacity of the population increases the invasion speed also increases, approaching the deterministic speed (Fig. 3). The stochastic model does therefore behave like the deterministic model when the population is big enough.

We find the same pattern as in the deterministic model in that when the dispersal abilities of the disperser and establisher are similar but the population growth rate of the establisher is much higher than that of the disperser the invasion follows the speed of the establisher (Fig 3a). Also when the population growth rates of each morph are similar but the dispersal rate of the disperser is much higher than the dispersal rate of the establisher the invasion follows the speed of the disperser (Fig 3b). In the case where there is a big difference between the two phenotypes in terms of both their dispersal and establishment abilities, we find that at the lower carrying capacities the invasion follows the speed of the faster morph. However, there is a threshold carrying capacity where the invasion starts to be faster than the monomorphic speed, and as the carrying capacity increases further this increases to approach the anomalous deterministic speed (Fig. 3c).

**Figure 4:**
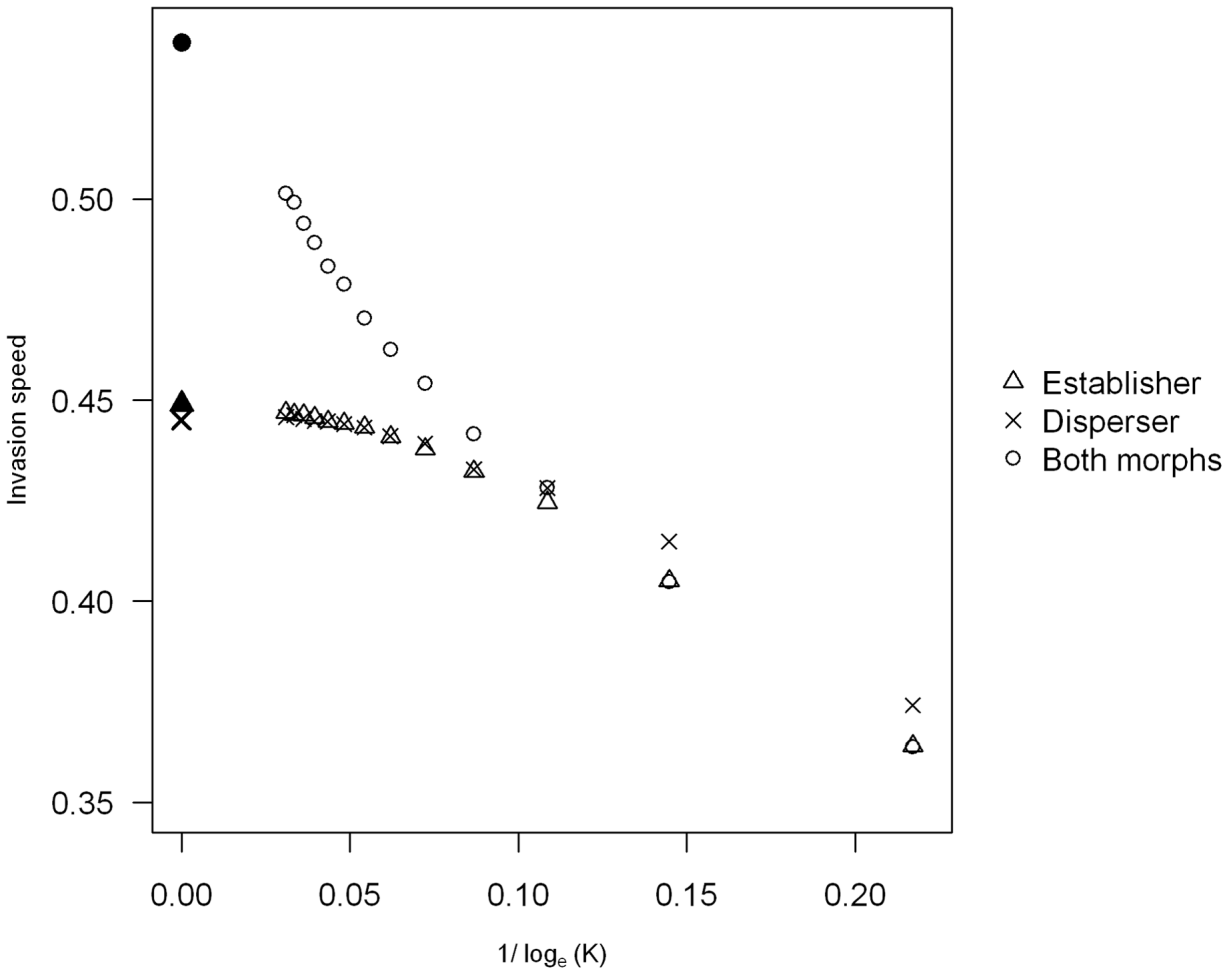
Comparison of invasion speeds with different values of 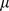. The parameter values are the same as in Fig 3(c) with the value of 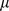 varied from 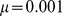 (crosses), 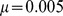 (diamonds), 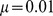 (circles), 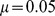 (plus) and 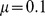 (triangles point down). The filled circle represents the polymorphic deterministic speed which is the same for all mutation rates. The triangles point up represent the fastest single morphs speed, which here is the establisher, with the filled triangle the deterministic speed.

We investigated how the mutation rate affects the threshold value for anomalous invasion speeds to occur for the case observed in Fig. 3c. We find that the higher the mutation rate the lower the threshold carrying capacity is for anomalous invasion speeds to occur (Fig. 4). High mutation rates (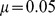 and 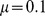) result in anomalous invasion speeds even when the carrying capacity is very low (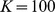). Smaller mutation rates (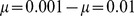) do not result in anomalous invasion speeds until the carrying capacity is much higher. This is in contrast to both the continuous and discrete deterministic models where we found that mutation rate had a negligible effect on the invasion speed. This could be because a higher mutation rate allows the morphs to keep up with each other better at the front of the invasion wave. A higher mutation rate therefore means that there are more of the morph that is at lower density present at the invasion front, in this case the disperser morph, and so there are more good dispersers along with the good establishers present at the front which results in faster invasions particularly when the carrying capacity is lower.

**Figure 5:**
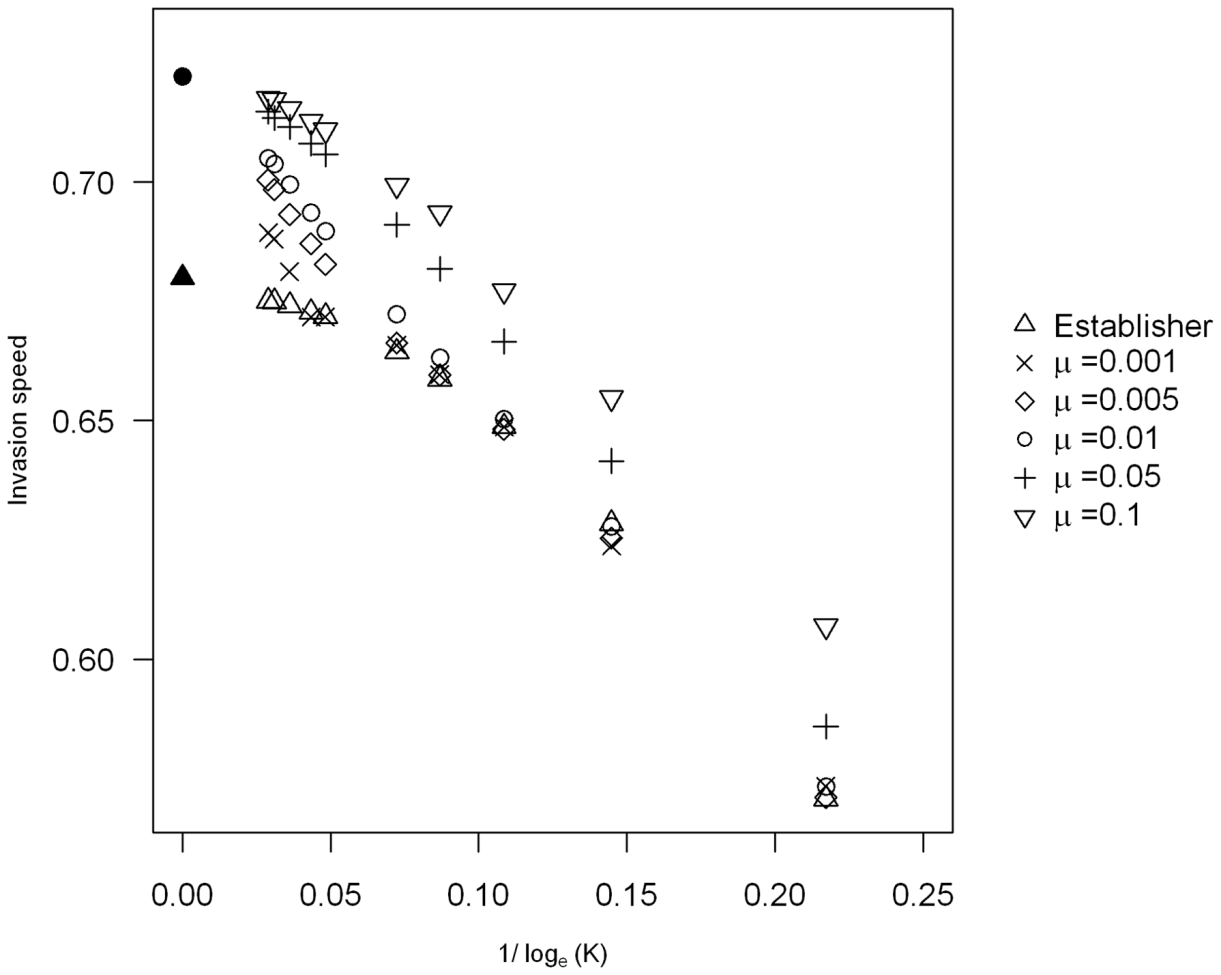
Anomalous invasion speeds when individual morph speeds are similar. The triangles represents the establisher morphs speed, the crosses the disperser morphs speed and the circles the invasion speed when both morphs are present. Parameter values used were 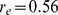, 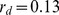, 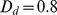, 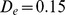, 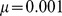 and 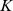 ranges from 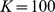 to 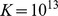.

However, we found that anomalous speeds are much more likely to be observed if the monomorphic speeds of either morph are similar to each other. For 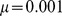 the lowest mutation rate investigated, we find that anomalous speeds can be observed from a carrying capacity of 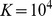 (Fig 5) whereas for the parameter values used in Fig 3 anomalous speeds were not observed at this mutation rate until 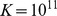. Therefore we find that if one morph has a faster invasion speed than the other, when both morphs are present high carrying capacities are required for anomalous speeds to be observed, however, if the individual morphs speeds are similar then when both morphs are present anomalous speeds are also found at low carrying capacities.

## Discussion

We have investigated the effect of the presence of two dispersal phenotypes on a species invasion speed in both a deterministic and stochastic model in discrete time and space. We found that if the morphs differ in both their dispersal ability and growth rate, then the invasion speed can be faster than the speed of either morph on its own. We have shown that this effect persists when demographic stochasticity is added into the model, although only when either the carrying capacity of the population is high enough or when the mutation rate between morphs is big. In many cases, an extremely large carrying capacity is required for anomalous speeds to be observed (e.g. about 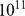 for the parameters in Fig. 3). However, if the two morphs have similar monomorphic invasion speeds then anomalous speeds are observed already at much smaller carrying capacities (e.g. about 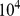 for the parameters in Fig. 5).

Our work agrees with previous studies that adding demographic stochasticity results in slower invasion speeds, with the deterministic speed predicting an upper bound for the stochastic speed [15], [16], [19]. Travis *et al*. [20] showed that the greater the amount of stochasticity the bigger the difference in the speeds predicted by their deterministic and stochastic models. Our results display a similar pattern, as we have shown that the stochastic speed tends towards the deterministic one when the degree of demographic stochasticity is decreased, in our case by increasing the carrying capacity of the population.

Since the wave speed in the deterministic limit is correctly predicted by the linear conjecture (see Fig. 2), i.e. by the low-density behaviour in the leading edge of the front, it is reasonable to expect that both morphs need to be present in high enough numbers in the leading edge in order to observe cooperative behaviour such as anomalous speeds. Our observation that higher carrying capacities and/or mutation rates make anomalous speeds more likely is consistent with this idea, because both of these conditions will boost the density of whichever morph is rarer in the leading edge. We have explored numerically the density profiles in order to understand the conditions under which anomalous speeds occur in stochastic models (see Appendix S4 in File S1). We do indeed find that anomalous speeds only occur when there is mutation between good establishers and good dispersers which are present at high densities in the leading edge of the invasion (see Fig. S1 and S2 in File S1), suggesting that these are necessary conditions for this emergent phenomenon. However, we also find in some cases that anomalous speeds do not occur even when both morphs have very high densities at the leading edge (see Fig. S1a in File S1). While it is clear that anomalous speeds arise from the dispersal ability of one morph and the establishment ability of the other, a simple quantitative criterion to predict exactly when we should observe anomalous invasion speeds in the stochastic model has so far elluded us.

At low mutation rates the carrying capacity required for anomalous polymorphic speeds is typically high–higher than the carrying capacity of many species. For example, despite some species exhibiting differences in their establishment and dispersal abilities, such as the western bluebird, *Sialia mexicana*, which has morphs with different dispersal behaviours [24], and flowering plants which exhibit seed polymorphism [25], these examples will not have populations with large enough carrying capacities to observe anomalous speeds. For low mutation rates the carrying capacity needs to be in the order of 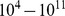 for faster speeds to be observed. Considering demographic stochasticity does therefore rule out a large number of species for which deterministic models would predict anomalous invasion speeds.

There are, however, species that have very large populations, in particular we may realistically expect to find populations with carrying capacities in the order of 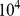 and so we may observe anomalous invasion speeds for species such as these. For example, many insect species exhibit trade-offs in their establishment and dispersal abilities through wing polymorphism (reviewed in [26], [27]) and also have very large population densities. In particular we may expect to see anomalous invasion speeds in insects such as crickets [28], planthoppers [29] and aphids [30] to name a few. We may also see anomalous invasion speeds in other taxa, for example, microorganisms can have very large population sizes, and some species of protists have been found to have trade-offs in their dispersal and establishment abilities [31].

In this model we have assumed that dispersal ability trades-off with population growth rate. Although this is the case for some species, with for example more dispersive individuals of the speckled wood butterfly, *Pararge aegeria*, having lower fecundity [32], this trade-off is not always observed. Some species show a positive relationship between dispersal and population growth rate, for example, more dispersive Glanville fritillary butterflies, *Melitaea cinxia*, have been found to invest more in reproduction [33], [34] and more dispersive cane toads, *Bufo marinus*, have been shown to have faster growth rates [35]. It has also been suggested that dispersal and reproductive rate may not directly trade-off against one another and may instead trade-off against other traits [36]. The anomalous speeds that we found using this model are unlikely to be observed in species where there is no trade-off in dispersal and establishment ability.

We have modelled the invasion of a polymorphic species on a homogenous landscape which is not very realistic. Introducing spatial heterogeneity into the model would more realistically reflect the natural environment and so would be a natural extension to this model. We are also interested in whether dispersal polymorphism resulting in anomalous invasion speeds can help a species to keep up with the rate of climate change. Our next step in this research will therefore be to explicitly model a range expansion as a result of a shifting climate.

We have shown that the presence of two phenotypes can lead to unexpected results for the speed of biological invasions. Our results reveal that demographic stochasticity can slow invasions, however, this is dependent on the carrying capacity of the population and the mutation rate between morphs being high enough. We hope that our results motivate further research into understanding the difference between deterministic and stochastic models and the implications that anomalous speeds have for predicting the rate of range expansions.

## Supporting Information

File S1 **Appendices.**
(PDF)

## References

[1] Chen IC, Hill JK, Ohlemüller R, Roy DB, Thomas CD (2011) Rapid range shifts of species associated with high levels of climate warming. Science 333: 1024–1026. 21852500 10.1126/science.1206432

[2] Parmesan C, Yohe G (2003) A globally coherant fingerprint of climate change impacts across natural systems. Nature 421: 37–42. 12511946 10.1038/nature01286

[3] Root TL, Price JT, Hall KR, Schneider SH, Rosenzweigk C, et al (2003) Fingerprints of global warming on wild animals and plants. Nature 421: 57–60. 12511952 10.1038/nature01333

[4] Hickling R, Roy DB, Hill JK, Fox R, Thomas CD (2006) The distributions of a wide range of taxonomic groups are expanding polewards. Global Change Biology 12: 450–455.

[5] Sakai AK, Allendorf FW, Holt JS, Lodge DM, Molofsky J, et al (2001) The population biology of invasive species. Annual Review of Ecology and Systematics 32: 305–332.

[6] Ziska LH, Blumenthal DM, Runion GB, Hunt ER, Diaz-Soltero H (2011) Invasive species and climate change: an agronomic perspective. Climate Change 105: 13–42.

[7] Gurevitch J, Padilla DK (2004) Are invasive species a major cause of extinctions? Trends in Ecology and Evolution 19: 470–474. 16701309 10.1016/j.tree.2004.07.005

[8] Fisher RA (1937) The wave of advance of advantageous genes. Annals of Eugenics 7: 355–369.

[9] Skellam JG (1951) Random dispersal in theoretical populations. Biometrika 331 38: 196–218. 14848123

[10] Hastings A, Cuddington K, Davies KF, Dugaw CJ, Elmendorf S, et al (2005) The spatial spread of invasions: new developments in theory and evidence. Ecology Letters 8: 91–101.

[11] Jongejans E, Skarpaas O, Shea K (2008) Dispersal, demography and spatial population models for conservation and control management. Perspectives in Plant Ecology Evolution and Systematics 9: 153–170.

[12] Weinberger HF, Lewis MA, Li BT (2007) Anomalous spreading speeds of cooperative recursion systems. Journal of Mathematical Biology 55: 207–222. 17318629 10.1007/s00285-007-0078-6

[13] Elliott EC, Cornell SJ (2012) Dispersal polymorphism and the speed of biological invasions. PLoS One 7: e40496. 22911701 10.1371/journal.pone.0040496 PMC3401290

[14] Kot M, Medlock J, Reluga T, Walton DB (2004) Stochasticity, invasions, and branching random walks. Theoretical Population Biology 66: 175–184. 15465119 10.1016/j.tpb.2004.05.005

[15] Mollison D (1991) Dependence of epidemic and population velocities on basic parameters. Mathematical Biosciences 107: 255–287. 1806118 10.1016/0025-5564(91)90009-8

[16] Lewis MA (2000) Spread rate for a nonlinear stochastic invasion. Journal of Mathematical Biology 41: 430–454. 11151707 10.1007/s002850000022

[17] Lewis MA, Pacala S (2000) Modeling and analysis of stochastic invasion processes. Journal of Mathematical Biology 41: 387–429. 11151706 10.1007/s002850000050

[18] Clark JS, Lewis M, Horvath L (2001) Invasion by extremes: Population spread with variation in dispersal and reproduction. American Naturalist 157: 537–554. 10.1086/319934 18707261

[19] Snyder RE (2003) How demographic stochasticity can slow biological invasions. Ecology 84: 1333–1339.

[20] Travis JMJ, Harris CM, Park KJ, Bullock JM (2011) Improving prediction and management of range expansions by combining analytical and individual-based modelling approaches. Methods in Ecology and Evolution 2: 477–488.

[21] Lande R, Saether E, Enge BE (2003) Stochastic Population Dynamics in Ecology 356 and Conservation. Oxford: Oxford University Press.

[22] van Saarloos W (2003) Front propagation into unstable states. Physics Reports-Review Section of Physics Letters 386: 29–222.

[23] R Development Core Team (2011) R: A Language and Environment for Statistical Computing. Vienna, Austria.

[24] Duckworth RA (2008) Adaptive dispersal strategies and the dynamics of a range expansion. American Naturalist 172: S4–S17. 10.1086/588289 18554143

[25] Sorensen AE (1978) Somatic polymorphism and seed dispersal. Nature 276: 174–176.

[26] Zera AJ, Denno RF (1997) Physiology and ecology of dispersal polymorphism in insects. Annual Review of Entomology 42: 207–230. 10.1146/annurev.ento.42.1.207 15012313

[27] Guerra PA (2011) Evaluating the life-history trade-off between dispersal capability and reproduction in wing dimorphic insects: a meta-analysis. Biological Reviews 86: 813–835. 21199288 10.1111/j.1469-185X.2010.00172.x

[28] Roff DA, Tucker J, Stirling G, Fairbairn DJ (1999) The evolution of threshold traits: effects of selection on fecundity and correlated response in wing dimorphism in the sand cricket. Journal of Evolutionary Biology 12: 535–546.

[29] Langellotto GA, Denno RF, Ott JR (2000) A trade-off between ight capability and reproduction in males of a wing-dimorphic insect. Ecology 81: 865–875.

[30] Braendle C, Davis GK, Brisson JA, Stern DL (2006) Wing dimorphism in aphids. Heredity 97: 192–199. 16823401 10.1038/sj.hdy.6800863

[31] Fjerdingstad EJ, Schtickzelle N, Manhes P, Gutierrez A, Clobert J (2007) Evolution of dispersal and life history strategies – Tetrahymena ciliates. BMC Evolutionary Biology 7. 10.1186/1471-2148-7-133 PMC1997130 17683620

[32] Hughes CL, Hill JK, Dytham C (2003) Evolutionary trade-offs between reproduction and dispersal in populations at expanding range boundaries. Proceedings of the Royal Society of London Series B-Biological Sciences 270: S147–S150. 10.1098/rsbl.2003.0049 PMC1809962 14667365

[33] Hanski I, Saastamoinen M, Ovaskainen O (2006) Dispersal-related life-history trade-offs in a buttery metapopulation. Journal of Animal Ecology 75: 91–100. 16903046 10.1111/j.1365-2656.2005.01024.x

[34] Saastamoinen M (2007) Mobility and lifetime fecundity in new versus old populations of the Glanville fritillary buttery. Oecologia 153: 569–578. 17566782 10.1007/s00442-007-0772-5

[35] Phillips BL (2009) The evolution of growth rates on an expanding range edge. Biology Letters 5: 802–804. 19605384 10.1098/rsbl.2009.0367 PMC2827979

[36] Phillips BL, Brown GP, Shine R (2010) Life-history evolution in range-shifting populations. Ecology 91: 1617–1627. 20583704 10.1890/09-0910.1

