# Correction: Are Anomalous Invasion Speeds Robust to Demographic Stochasticity?

**DOI:** 10.1371/annotation/6b6a44ac-2e3d-4677-b9c8-2bc4cfb20c7e

**Published:** 2013-07-18

**Authors:** Elizabeth C. Elliott, Stephen J. Cornell

Figures 4 and 5 were incorrectly switched. The image appearing as Figure 4 belongs with the title and legend appearing with Figure 5, and the image appearing as Figure 5 belongs with the title and legend appearing with Figure 4. The titles and legends themselves are in correct order. 

