# Supplementary material for: Are Anomalous Invasion Speeds Robust to Demographic Stochasticity?
Source: PLoS One. 2013 Jul 16;8(7):e67871. doi: 10.1371/journal.pone.0067871 (PMC3713005; doi:10.1371/journal.pone.0067871)
Supplement: File S1 — Appendices. (PDF) [file pone.0067871.s001.pdf]

File S1: Appendices

Elizabeth C. Elliott   Stephen J. Cornell

Appendix S1: Reparameterisation of model

We wish to reduce the number of parameters in the model and make it so that the model is more easily comparable to our previous work [1]. To do this we first look at the nonspatial single morph model which is given by:

$$N_i(t+1) = (1+b)N_i(t)\exp(-m_i^0 - m_i^1(1+b)N_t)$$

For the purpose of this model we want the carrying capacity K to be fixed by the equilibrium population density. We therefore calculate the equilibrium density N_i^* by setting $N_i(t+1) = N(t)$ which gives the equilibrium to be $N_i^* = \frac{\log(1+b)-m_i^0}{m_i^1(1+b)}$ and then set $K = N_i^*$.

The nonspatial version of the continuous model in [1] is given by $\frac{dN_i}{dt} = r_i N_i(1 - \frac{N_i}{K})$. To relate the population growth rate term to the parameters in the discrete model we assume that when N_i is small we get exponential growth in one time step. We can therefore assume that when $N_i << K$ we have $N_t = N_0 e^{r_i t}$ where N_0 is the initial population size and t is time. In the discrete model when $N_i << K$ we have that $N_t = RtN_0$ where $R = (1+b)e^{-m_i^0}$. We can therefore relate the continuous and discrete models by $R = e^{r_i}$, which gives $r_i = \log(1+b) - m_i^0$.

In the full spatial model the parameter D_i is defined in the same way as in [1] and so parameter values used in the two models are directly comparable.

Appendix S2: Deterministic limit of the stochastic model when $K \to \infty$

We discuss here the model in one spatial dimension, though exactly the same discussion would apply in higher dimensions. The stochastic version of the model can be written in terms of Poisson- and Binomially-distributed random variables as follows:

$$\begin{aligned}
B_i(t,x) &= N_i(t,x) + X_i(t,x)\\
M_i(t,x) &= Y_i(t,x)\\
N_i(t+1,x) &= M_i(t,x) - \tilde{N}_i(t,x) + \frac{1}{2}\tilde{N}_i^-(t,x+1) + \frac{1}{2}\tilde{N}_i^+(t,x-1),
\end{aligned}$$

where

$$\begin{aligned}
X_i(t,x) &\sim \text{Poi}\left(b\left\{(1-\mu)N_i(t,x) + \mu N_j(t,x)\right\}\right)\\
Y_i(t,x) &\sim \text{Bin}(B_i(t,x), P_i(t,x))\\
\tilde{N}_i(t,x) &\sim \text{Bin}(M_i(t,x), D_i)\\
\tilde{N}_i^+(t,x) &\sim \text{Bin}(\tilde{N}_i(t,x), \frac{1}{2})\\
\tilde{N}_i^-(t,x) &= \tilde{N}_i(t,x) - \tilde{N}_i^+(t,x)
\end{aligned}$$

and P_i is defined in Eqn. (2) in the main body of the paper. We now define rescaled variables

$$n_i(t,x) = \frac{N_i(t,x)}{K} \tag{A1}$$

$$c_i(t,x) = \frac{B_i(t,x)}{K} \tag{A2}$$

$$l_i(t,x) = \frac{M_i(t,x)}{K}, \tag{A3}$$

where $K = \frac{(\log b - m_i^0)}{(1+b)m_i^1}$, in terms of which the equations can be written as

$$c_i(t,x) = n_i(t,x) + b\{(1-\mu)n_i(t,x) + \mu n_j(t,x)\} + \xi_i(t,x) \tag{A4}$$

$$l_i(t,x) = c_i(t,x)p_i(t,x) + \zeta_i(t,x) \tag{A5}$$

$$\begin{aligned} n_i(t+1,x) =& (1-D_i)l_i(t,x) + \frac{D_i}{2}l_i(t,x+1) + \frac{D_i}{2}l_i(t,x-1) + \\ & \nu^+(t,x-1) + \nu^-(t,x+1) - \nu^+(t,x) - \nu^-(t,x), \end{aligned} \tag{A6}$$

where

$$\begin{aligned} p_i &= \exp\left(-m_i^0 - \frac{(\log b - m_i^0)}{1+b}\left(c_i(t,x) + c_j(t,x)\right)\right) \\ \xi_i &= \frac{1}{K}\left(X_i(t,x) - b\{(1-\mu)N_i(t,x) + \mu N_j(t,x)\}\right) \\ \zeta_i &= \frac{1}{K}\left(Y_i(t,x) - P_i(t,x)B_i(t,x)\right) \\ \nu_i^{\pm} &= \frac{1}{K}\left(\tilde{N}_i^{\pm} - \frac{1}{2}M_i(t,x)D_i\right). \end{aligned}$$

From the properties of Poisson and Binomial distributions, the mean and variances of the noise terms ξ_i, ζ_i, and $\nu_i^{\pm}$ are

$$\begin{aligned} E(\xi_i(t,x)) &= 0 = E(\zeta_i) = E(\nu_i^{\pm}) \\ \mathrm{Var}(\xi_i(t,x)) &= \frac{b}{K}\{(1-\mu)n_i(t,x) + \mu n_j(t,x)\} \\ \mathrm{Var}(\zeta_i(t,x)) &= \frac{p_i(t,x)(1-p_i(t,x))c_i(t,x)}{K} \\ \mathrm{Var}(\nu_i^{\pm}(t,x)) &= \frac{D_i(1-D_i)l_i(t,x)}{4K} \end{aligned}$$

The variance of these noise terms ξ_i, ζ_i, and $\nu_i^{\pm}$ is proportional to K^{-1}, and therefore vanishes when $K \to \infty$ provided n_i remains finite in this limit. In that case, equations (A4–A6) converge to the deterministic equations

$$c_i(t,x) = n_i(t,x) + b\{(1-\mu)n_i(t,x) + \mu n_j(t,x)\} \tag{A7}$$

$$l_i(t,x) = c_i(t,x)p_i(t,x) \tag{A8}$$

$$n_i(t+1,x) = (1-D_i)l_i(t,x) + \frac{D_i}{2}l_i(t,x+1) + \frac{D_i}{2}l_i(t,x-1), \tag{A9}$$

which are exactly the same equations that would be obtained by applying the rescaling (A1–A3) to the original deterministic model (Eqns. (1–5) in the main body of the paper). Note that the parameter K does not appear in Eqns. (A7–A9), so that, if we start with an initial condition where c_i is of order 1, then it will remain of order 1. Therefore, the stochastic model converges to the deterministic model when $K \to \infty$.

Appendix S3: Calculation of invasion speed

We used the front propagation method of van Saarloos [2] to calculate the invasion speed. Here we present the results of this method for the case where both morphs are present; we also calculated the invasion speed using this method for the single morph cases but we do not present these results as they are similar to the more complex two morph case. The general system of spatially uniform equations has two equilibria: an unstable extinction state where $(N_e, N_d) = (0, 0)$, and a stable coexistence state which we will denote by (N_e^*, N_d^*). The front propagation method involves linearising the equations about the unstable steady state, which gives:

$$\begin{aligned} N_e(t,x) =& e^{-m_e^0}[(1+b-\mu b)[(1-D_e)N_e(t,x) + \frac{1}{2}D_e(N_e(t,x+1)+N_e(t,x-1))] \\ &+ \mu b[(1-D_e)N_d(t,x) + \frac{1}{2}D_e(N_d(t,x+1)+N_d(t,x-1))]] \end{aligned} \tag{A10}$$

$$\begin{aligned} N_d(t,x) =& e^{-m_d^0}[(1+b-\mu b)[(1-D_d)N_d(t,x) + \frac{1}{2}D_d(N_d(t,x+1)+N_d(t,x-1))] \\ &+ \mu b[(1-D_d)N_e(t,x) + \frac{1}{2}D_d(N_e(t,x+1)+N_e(t,x-1))] \end{aligned} \tag{A11}$$

Here we are assuming that the speed we calculate using the linear system (A10) and (A11) also applies to the nonlinear system (1-4) in the main body of the paper. This linear speed is known to be a lower bound of the invasion speed but it is not always exact [3]. We therefore compare these results to those we found from the numerical simulations to check that this linear conjecture is valid.

Using these linearised equations, and following [2], we substitute $\begin{pmatrix} N_e \\ N_d \end{pmatrix} \propto \begin{pmatrix} n_e \\ n_d \end{pmatrix} \exp(-i\omega(k)t + ikx)$ where $\omega(k)$ is the dispersion relation of Fourier modes of the linearised equations (A10), (A11) and k is the spatial wavenumber. This gives the equations:

$$[e^{-m_e^0}[(1+b-\mu b)(1-D_e+D_e\cos k)] - e^{-i\omega(k)}]n_e + e^{-m_e^0}\mu b(1-D_e+D_e\cos k)n_d = 0$$

$$e^{-m_d^0}\mu b(1-D_d+D_d\cos k)n_e + [e^{-m_d^0}[(1+b-\mu b)(1-D_d+D_d\cos k)] - e^{-i\omega(k)}]n_d = 0$$

This leads to an eigenvalue problem, with solutions

$$\omega(k) = i[log[(1+b-\mu b)[e^{-m_e^0}(1-D_e+D_e\cos k) + e^{-m_d^0}(1-D_d+D_d\cos k)] \pm R] - \log 2]$$

where $R^2 = [(1+b)^2 - 2\mu b(1+b)][e^{-m_e^0}(1-D_e+D_e\cos k) - e^{-m_d^0}(1-D_d+D_d\cos k)]^2 + \mu^2 b^2 [e^{-m_e^0}(1-D_e+D_e\cos k) + e^{-m_d^0}(1-D_d+D_d\cos k)]^2$. This implies

$$\frac{d\omega(k)}{dk} = i\left(\frac{-(1+b-\mu b)(e^{-m_e^0}D_e\sin k + e^{-m_d^0}D_d\sin k) \pm \frac{dR}{dk}}{(1+b-\mu b)[e^{-m_e^0}(1-D_e+D_e\cos k) + e^{-m_d^0}(1-D_d+D_d\cos k)] \pm R}\right)$$

We then calculate the wave speed by finding k^*, where k^* is the linear spreading point [2], such that

$$\frac{d\omega(k^*)}{dk^*} = \frac{\Im\omega(k^*)}{\Im k^*} \tag{A12}$$

where $\Im$ denotes the imaginary part. These equations represent a biological invasion so n_e and n_d cannot be negative, so we can deduce that k^* is purely imaginary. We can then assume $k^* = iq$ with q real, which we substitute into (A12). The realised wavespeed is obtained by finding the real solution q that corresponds to the largest speed $v = \frac{\Im\omega(iq)}{q}$ [2]. Substituting into Eqn. (A12) gives an equation which cannot be solved analytically. We therefore solve this numerically and then calculate the invasion speed v using this value of q.

Appendix S4: Population density at invasion front

Typically we find that when anomalous speeds occur the establisher morph is at higher densities just behind the invasion front but in the leading edge the disperser morph is at higher density (Fig S.1). This is in contrast to the case where the invasion follows the speed of either the establisher or disperser as here whichever is the faster morph in isolation is at higher density in the leading edge (Fig S.2). As can be seen in Fig. S.1 the qualitative pattern is that as the carrying capacity gets higher and so we get higher densities of both morphs at the invasion front we observe anomalous speeds. For anomalous speeds to occur it is therefore important for both establishers and dispersers to be present at high enough densities at the invasion front. However this is not always the case, as Fig. S.1(a) shows there are some cases where as we increase the carrying capacity and so there is a higher density at the invasion front we do not observe anomalous speeds, until the carrying capacity becomes even bigger. It is therefore difficult to determine what exact conditions are required for these speeds and as yet we have been unable to do so. As can be seen though the phenomenon described above, where there is a switch in the morph that is at higher density at the invasion front, does seem to be a necessary condition for anomalous speeds to occur although it does not always result in them.

References

[1] Elliott EC, Cornell SJ (2012) Dispersal polymorphism and the speed of biological invasions. PLoS One 7: e40496.

[2] van Saarloos W (2003) Front propagation into unstable states. Physics Reports-Review Section of Physics Letters 386: 29-222.

[3] Weinberger HF, Lewis MA, Li BT (2002) Analysis of linear determinacy for spread in cooperative models. Journal of Mathematical Biology 45: 183-218.

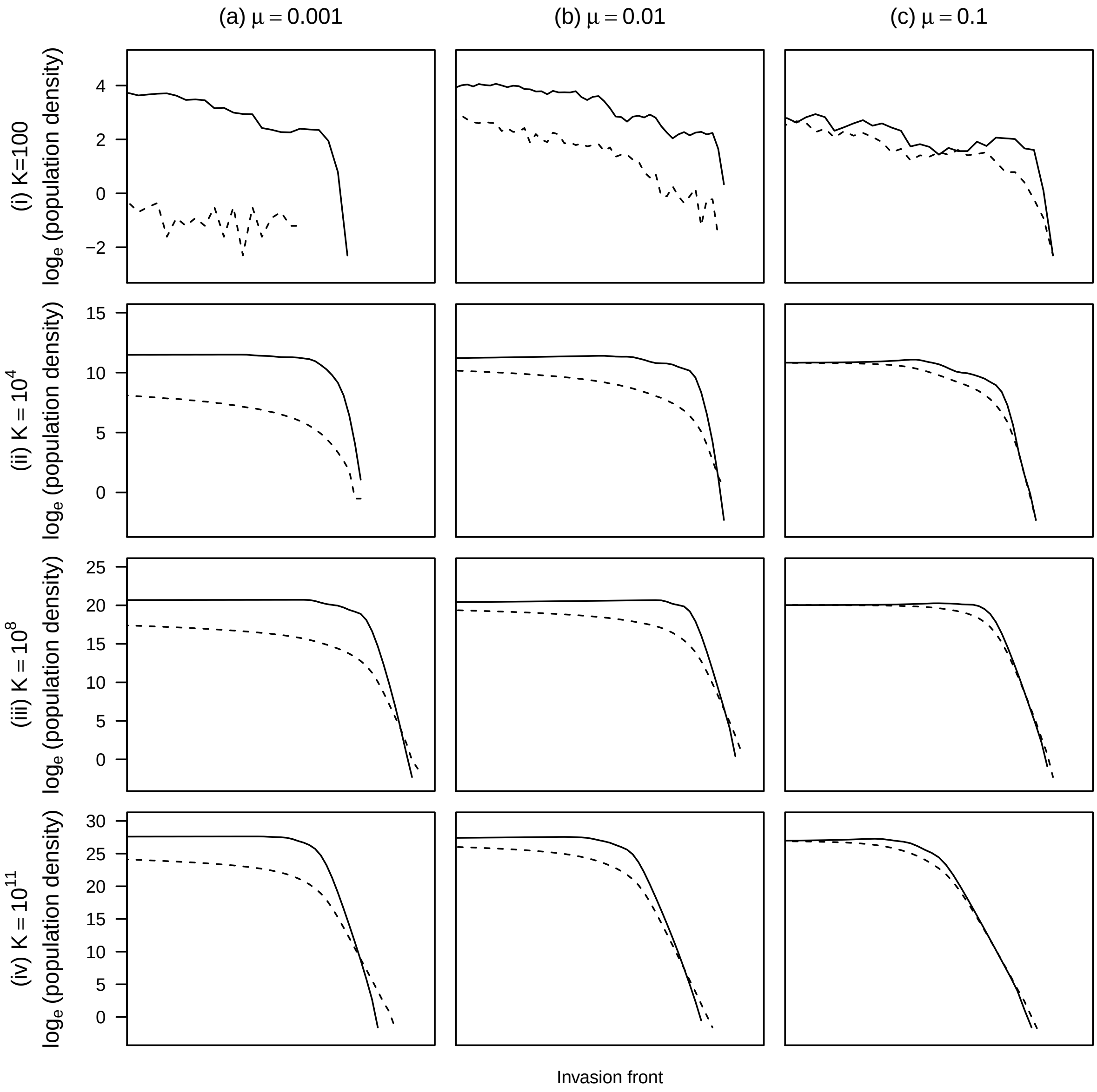

Figure S. 1: Comparison of population densities at the invasion front for the case where anomalous speeds occur. We have plotted the log(population density) for the front 50 cells occupied by the population at time $t = 8000$, where the solid line is the establisher morph and the dashed line the disperser morph. In (a) μ=0.001 and here anomalous speeds were only found for carrying capacity (iv), however the disperser is at a higher density from (iii), see Fig. 4 for details of when we observe anomalous invasion speeds. In (b) μ=0.01 and anomalous speeds occur from carrying capacity (iii) which is the first time the disperser is at higher density at the invasion front. In (c) anomalous speeds occur from (i) and the disperser morph is at higher density for all carrying capacities. Parameter values used are the same as in Fig. 4.

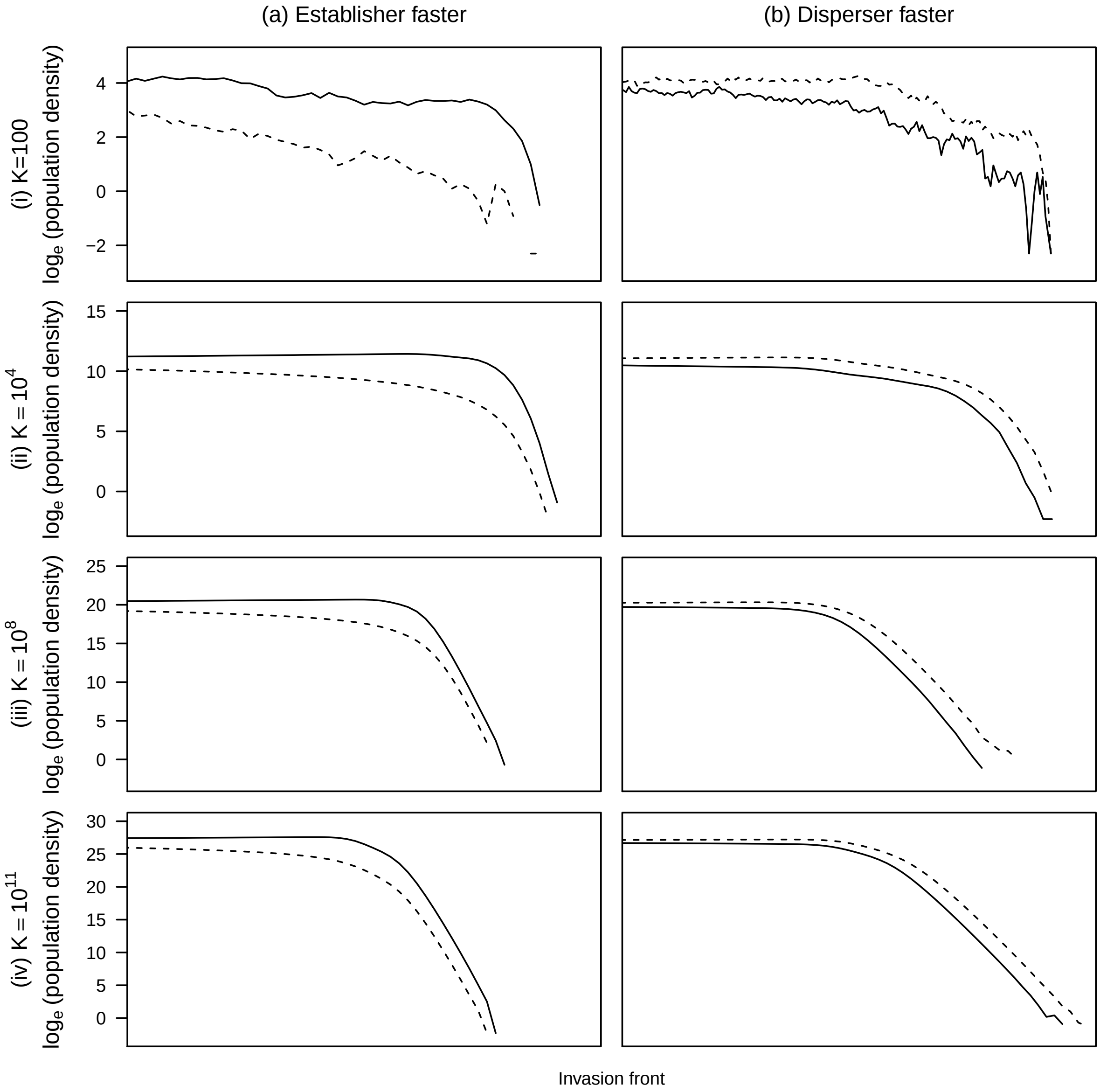

Figure S. 2: Comparison of population densities at the invasion front, where in (a) when both morphs are present the invasion follows the speed of the establisher morph and in (b) the invasion follows the speed of the disperser morph. We have plotted the log(population density) for the front 50 cells occupied by the population at time $t = 8000$, where the solid line is the establisher morph and the dashed line the disperser morph. In (a) the establisher morph is at higher density at the invasion front for all carrying capacities and in (b) the disperser morph is at higher density for all carrying capacities. Parameter values used are the same as in Fig. 3.
